# Simultaneous Extraction and Determination of Lignans from *Schisandra chinensis* (Turcz.) Baill. via Diol-Based Matrix Solid-Phase Dispersion with High-Performance Liquid Chromatography

**DOI:** 10.3390/molecules28186448

**Published:** 2023-09-05

**Authors:** Yinpeng Wang, Jingbo Zhu, Xinxin Du, Yumei Li

**Affiliations:** 1 School of Food Science and Technology, Dalian Polytechnic University, Dalian 116034, China; 18308320017026@xy.dlpu.edu.cn (Y.W.); dxx_dpu@126.com (X.D.); 2 Department of Clinical Pharmacy and Traditional Chinese Medicine Pharmacology, School of Pharmaceutical Sciences, Changchun University of Chinese Medicine, Changchun 130117, China

**Keywords:** *Schisandra chinensis* (Turcz.) Baill., lignan, matrix solid-phase dispersion, diol-functionalized silica, quantification

## Abstract

The quality of *Schisandra chinensis* (Turcz.) Baill. (*S. chinensis*) is principally attributed to lignan compounds. In this paper, a simple and rapid strategy for simultaneous extraction and determination of 10 lignans from *S. chinensis* was established through matrix solid-phase dispersion (MSPD) assisted by diol-functionalized silica (Diol). The experimental parameters for MSPD extraction were screened using the response surface methodology (RSM). Diol (800 mg) was used as a dispersant and methanol (MeOH, 85%, *v*/*v*) as an eluting solvent (10 mL), resulting in a high extraction efficiency. MSPD extraction facilitated the combination of extraction and purification in a single step, which was less time-consuming than and avoided the thermal treatment involved in traditional methods. The simultaneous qualification and quantification of 10 lignans was achieved by combining MSPD and high-performance liquid chromatography (HPLC). The proposed method offered good linearity and a low limit of detection starting from 0.04 (schisandrin C) to 0.43 μg/mL (schisantherin B) for lignans, and the relative standard deviation (RSD, %) values of precision were acceptable, with a maximum value of 1.15% (schisantherin B and schisanhenol). The methodology was successfully utilized to analyze 13 batches of *S. chinensis* from different cultivated areas of China, which proved its accuracy and practicability in the quantitative analysis of the quality control of *S. chinensis*.

## 1. Introduction

*Schisandra chinensis* (Turcz.) Baill. (*S. chinensis*), a red-colored fruit with five flavor profiles (sour, bitter, sweet, spicy, and salty), was added to the list of health foods by the Ministry of Health of the People’s Republic of China in 2002 as it was increasingly used as a foodstuff and food additive [1,2]. Geographically, *S. chinensis* is mainly cultivated in the northeast of China, including Liaoning, Jilin, Heilongjiang, and Inner Mongolia, and is also distributed among Korea, Japan, and Russia [3,4]. *S. chinensis* has long been known as a valuable source of raw material for medicine with physiological effects, and has been widely used in clinical practice for the improvement of sleep [5,6], for its hepatoprotective properties [7], and for immune regulation [8,9]. In recent years, *S. chinensis* has also been extensively consumed as an essential source of nutritional and functional ingredients in food (jam, and beverages such as tea) [10,11].

*S. chinensis* is rich in phytonutrients with health benefits, with the classes of lignan, flavonoid, terpenoid, and polysaccharide compounds. In particular, lignans with a dibenzocyclooctadiene structure were recognized as the most significant class of bioactive ingredients. It has been reported that lignans such as schisandrol A and schisandrol B have hepatoprotective [12,13], anticancer [14,15], and neuroprotective functions [16]. Meanwhile, schisandrol A is one of the typical analytes of *S. chinensis* and considered as a quality control marker according to the Chinese Pharmacopeia 2020 [1]. In addition, a variety of other lignans, including schisandrin A, schisandrin B, schisandrin C, angeloylgomisin H, and gomisin J, possess a wide range of pharmacological activities like antioxidation [17,18,19], anti-inflammation [20,21], antifibrosis [22,23], cancer prevention [24], and metabolic regulation [25,26]. Consequently, the complete extraction and precise analysis of lignans via a simple and efficient strategy leads to great benefits for the quality control and bioactive investigations of *S. chinensis*.

As of now, a series of techniques for determining multiple ingredients in *S. chinensis* have been applied using high-performance liquid chromatography (HPLC) [27], gas chromatography–mass spectrometry (GC-MS) [28], UPLC (UPLC-Q-TOF-MS) [29], and supercritical fluid chromatography (SFC) [30]. Due to the complexity of the matrix, the procedure for sample pretreatment is often perceived as an obstruction for analytical methods, and maximizing recovery of the analytes and minimizing the accompanying interferences through the appropriate extraction and clean-up procedures are perceived as critical steps in ensuring the efficiency of analytical procedures [31]. The conventional extraction procedures, including refluxing [32], ultrasonic [33], microwave-assisted [34], smashing tissue [35], and supercritical CO_2_ extraction [36], often require additional clean-up or concentration steps, which involve both time and solvent consumption. They may also lead to a reduction in the number of analytes due to heating, hydrolysis, or other mechanisms. These variables deeply affect the extraction efficiency of the analytes and contribute significantly to the total cost of the analysis.

Matrix solid-phase dispersion (MSPD), first proposed by Barker in 1989 [37], is a process that involves blending analytes with a polymeric phase bound to a solid support, and finally combining the crushing, extraction, separation, and purification processes in a single process based on their dissolution characteristics in the matrix [38]. In comparison with the conventional sample preparation methods, MSPD eliminates duplicate centrifugation, concentration, filtration, and re-extraction procedures, and avoids the thermal process. This would significantly reduce the quantity of solvent used and the time requirement for preparation-related manipulation, and eliminate degradation in the sample composition. In addition, the selection of the appropriate dispersant is one of the critical procedures in the extraction progress of MSPD [39]. Diol-functionalized silica (Diol), a hydrophilic silica dispersant (HILIC) bonded with the 1,2-dihydroxypropyl propyl ether functional group, has a unique retention property for polar components due to the moderate positive phase retention provided by the two hydroxyls on the silica surface [40]. Meanwhile, the additional hydrophobic effect is provided by the Diol bonded carbon chain, which has superior selectivity for nonpolar components and enables effective separation for low polar compounds on polar stationary phases [41,42]. For these reasons, Diol has been developed into commercial stationary phases, which are becoming increasingly popular for the extraction and separation of complex matrices, and are applied to extract coumarins and phenolic acids from *Angelicae pubescentic radix* [40], separate phospholipids in plasma and cell membranes [43,44], and separate flavanones from bee pollen [45]. These properties make Diol a valid dispersant in the MSPD method which can enhance the extraction efficiency of analytes with this method.

This study aimed to establish a Diol-based MSPD method for simultaneous extraction and determination of lignans in *S. chinensis* using high-performance liquid chromatography with a diode array detector (HPLC-DAD), which is a common instrument accessible in most routine laboratories. The effects of several parameters, including the type of dispersant, sample/dispersant ratio, elution solvent, concentration, and elution volume, were investigated and optimized with response surface methodology (RSM). Furthermore, the chosen Diol had specific interactions with the analytes and sharpened the sensitivity of these parameters. This proposed method can be effectively used for the pretreatment of raw *S. chinensis* materials or its processed products for quality control.

## 2. Results and Discussion

### 2.1. Validation of the Method

In this study, an HPLC-DAD analytical method coupled with Diol-based MSPD was established, optimized, and validated for the simultaneous determination of 10 lignans with the aim of achieving optimal chromatographic resolution. Analyte standard solutions allowed the construction of external calibration curves using peak area values as the response. In Table 1, the linearity of 10 analytes is constructed with the peak areas of the analytes (*Y*-axis) and concentration (*X*-axis), which ranged from 0.56 μg/mL (schisandrin C) to 175 μg/mL (schisandrol A), and the minimum value of R^2^ was 0.9991 (schisandrin C). The limit of detection (LOD) and limit of quantitation (LOQ), which were calculated as the minimum concentration of the analytes that yield the S/N ratios of 3 and 10, respectively, were in the range of 0.04 (schisandrin C) to 0.43 μg/mL (schisantherin B) and 0.49 (schisandrin C) to 2.07 μg/mL (schisantherin B), respectively.

In addition, reproducibility was assessed from the intraday (repeatability) and interday (intermediate precision) values using the relative standard deviation (RSD) of the peak area for each analyte at a certain concentration level. As shown in Table 1, the RSD values of repeatability ranged from 0.21% (schisandrin C) to 1.05% (schisanhenol). Regarding the intermediate precision, the RSD values were between 0.80% (schisandrin B) and 1.15% (schisantherin B and schisanhenol). In sum, the reliable performance and reproducibility of the method established were attained for all the analytes studied.

### 2.2. MSPD Extraction Procedure

#### 2.2.1. The Selection of Parameters in MSPD Procedure

To achieve the highest extraction yield of total lignans from *S. chinensis*, a series of experiments were performed to determine the preliminary parameters for MSPD extraction, including the type of dispersant and mass ratio to the sample, elution solvent, concentration, and elution volume.

**Figure 1:**
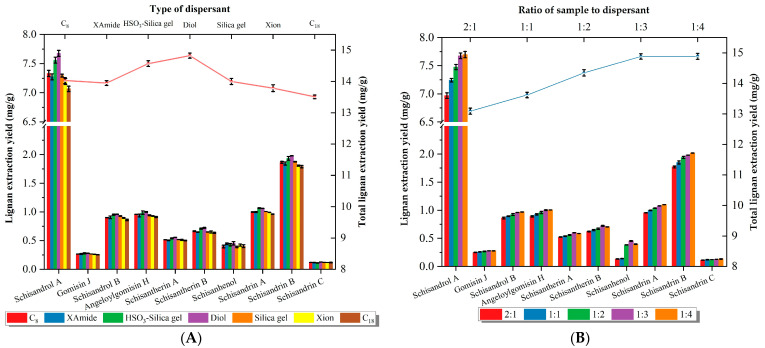

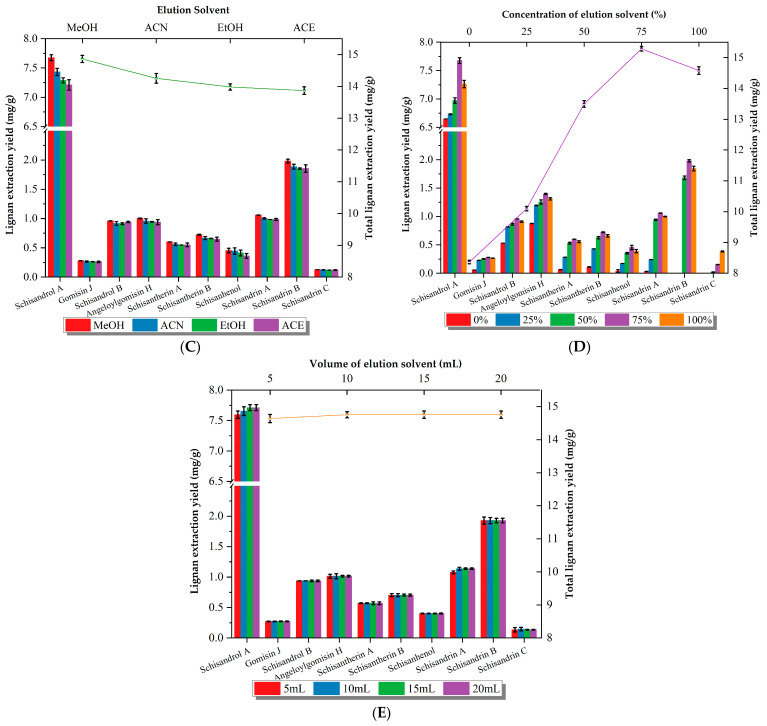
Effect of parameters on efficiency of 10 lignans. (**A**) Type of dispersant. (**B**) Ratio of sample to dispersant. (**C**) Elution solvent. (**D**) Concentration of elution solvent. (**E**) Volume of elution solvent. The bars represent the extraction yield of each lignan and the line–symbol plots represent the total lignan extraction yield.

The type of dispersant determined the high adsorption capacity and selectivity between the matrix and dispersant [46]. In this study, the extraction efficiencies of seven dispersants (silica gel, HSO_3_-silica gel, C_18_, C_8_, Xion, XAmide, and Diol) based on silica gel were investigated for the lignans of *S. chinensis*. Figure 1A shows that the extraction capacity of the seven dispersants for the target analytes varied, and Diol showed a superior extraction ability compared with the others. Synthesized with unmodified bare silica gel, Diol was bonded with 1,2-dihydroxypropyl propyl ether functional groups that provided additional hydrophobicity to low polar compounds, and showed smaller differences in selectivity and shorter retention times, which resulted in low retention of low polar substances represented by lignans in the Diol phase [42,47]. Additionally, the Diol phase used in the HILIC mode formed an aqueous enriched layer at the surface of the stationary phase, which was capable of adsorbing hydrophilic molecules (including polyphenols, proteins, and phospholipids) from extracted solutions of *S. chinensis* by hydrogen force bonding with the sample, while basically retaining nothing of the lignans and obtaining a high extraction efficiency [40,43,48]. Therefore, Diol was selected as the optimal dispersant for the subsequent extraction process.

Next, the mass ratio of *S. chinensis* sample to dispersant (2:1 to 1:4) with the quantity of dispersant varying from 100 to 800 mg was studied. The sample/dispersant ratio could affect the extraction efficiency since it not only determined the interface area between sample and dispersant, but also influenced the elution of the analytes [49]. As shown in Figure 1B, the extraction efficiency of 10 lignans significantly increased as the ratio rose from 2:1 to 1:3, and leveled off when it changed to 1:4. This was due to the appropriate amount of adsorbent, which produced a larger interface area and stronger intermolecular interactions. Therefore, the sample/dispersant ratio of 1:3 was adopted for further studies.

Then, a series of elution solvents including methanol (MeOH), acetonitrile (ACN), ethanol (EtOH), and acetone (ACE) were chosen to be investigated. According to the results in Figure 1C, MeOH revealed an advantage in extraction efficiency. Moreover, in Figure 1D, it can be seen that with the increase in MeOH concentration in the elution solvent (from a water solution to 75% MeOH-H_2_O), the retention factor of the solute decreased and the lignan extraction efficiency increased significantly. But, as the concentration continued to increase (from 75% MeOH-H_2_O to 100% MeOH), the extraction efficiency of the lignans was not satisfactory. And in Figure 1E, it can be seen that the extraction recoveries of the analytes were consistently slightly increased with the increase in the solution volume scale from 5 mL to 10 mL; it was not until 15 mL was reached that the extraction no longer increased. To sum up, the parameters of 10 mL and 75% MeOH-H_2_O were chosen for subsequent experiments.

#### 2.2.2. Optimization of MSPD Procedure with RSM

As a multivariate chemometric approach, RSM can visualize the interaction effects between process variables and responses [50]. Therefore, RSM was employed to obtain the optimal parameters of MSPD on the basis of mono-factor experimental results. The Box–Behnken design (BBD) was generally chosen as an optimization model through RSM [51]. The BBD matrices and experimental values for the total lignan extraction yield are presented in Table 2. After performing 17 runs, the empirical relationship between the response and independent variables was evaluated using the following equation:

(1) $$Y=14.4900+0.0850X_{1}+0.8013X_{2\;}+0.0838X_{3}+0.0400X_{1}X_{2}-\;0.0200X_{1}X_{3}-0.0625X_{2}X_{3}-0.1563X_{1}^{2}-0.9688X_{2}^{2}-0.1087X_{3}^{2}$$

where Y is the response value, and X_1_, X_2_, and X_3_ respectively correspond to the values of three independent variables: sample/dispersant ratio, solvent concentration, and elution volume.

**Figure 2:**
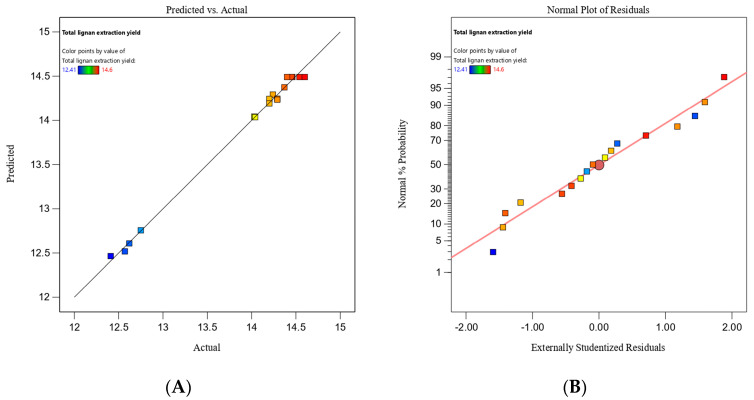
Graph of (**A**) actual values vs. predicted values for extraction yield with MSPD. (**B**) Normal % probability plot of internally studentized residuals.

The significance and adequacy of the model were analyzed by using the F-test (Table 3). From the analysis of variance (ANOVA) results, the model had a significantly high F-value (F = 182.02) and low *p*-value for the responses (*p* < 0.0001, less than 0.05), indicating the model terms were significant. The R^2^ was 0.9957, which suggested the high degree of fitness in this model. Furthermore, the lack of fit was 0.5462 (more than 0.05), indicating that the model reliably estimated the optimal experimental parameters. Furthermore, the diagnostic and correlation plots were assessed to recognize the significance of the obtained ANOVA [52]. Evaluation of the optimized design revealed (Figure 2A) that the actual values obtained from the research were basically close to a straight line, demonstrating a good correlation with the predicted values. Meanwhile, the normal plot of residuals was established to illustrate the accuracy of the optimized residuals. In Figure 2B, the normal probability distribution of the residuals is basically on a straight line, indicating that the predicted model is accurate. All these results revealed that the RSM model was suitable for precise estimations of the extraction conditions of lignans.

According to the results in Table 3, the most influential factor on MSPD extraction efficiency was the linear term of MeOH concentration (X_2_, *p* < 0.05), followed by the quadratic term of MeOH concentration (X_2_^2^, *p* < 0.05) and the quadratic term of the sample/dispersant ratio (X_1_, *p* < 0.05). The significant model terms with low *p*-values signified a more critical impact on the respective response variables. Thus, these data suggested that the MeOH concentration was a crucial variable for the extraction of total lignans by using MSPD.

#### 2.2.3. Analysis of Response Surface

**Figure 3:**
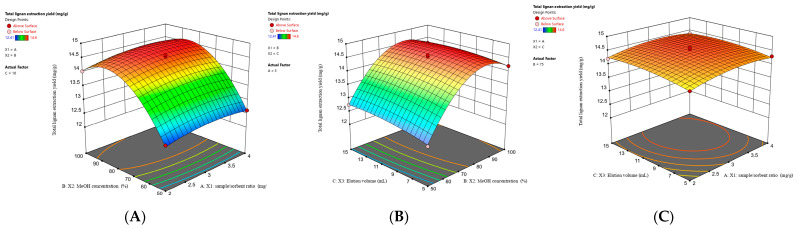
Response surface from Box–Behnken design (BBD) for the effect of independent variables on the extraction of target analytes. (**A**) Sample/dispersant ratio and MeOH concentration. (**B**) MeOH concentration and elution volume. (**C**) Elution volume and sample/dispersant ratio.

To provide a preferable visualization of the independent variables on the extraction yield, 3D response surface curves of this model were drawn in Figure 3. The response of the total lignan extraction yield served as a quality control marker associated with sample/dispersant ratio, MeOH concentration, and elution volume. As shown in Figure 3A, the response increased with increasing MeOH concentration, and the maximum extraction yield was obtained at 75%. Notably, when the concentration reached a higher level (100%), the response decreased slightly. This is due to the relatively low hydrophobicity of the tested polar lignans represented by schisandrol A and schisandrol B compared with other analytes resulting in a slight decline in the extraction yield under 100% MeOH. And no significant changes in the sample/dispersant ratio were observed. In Figure 3B, with the increase in MeOH concentration, the response of the extraction yield showed a trend of rising at first and then declining, but there was no obvious effect of volume on the response effect. The effects of elution volume and sample/dispersant ratio (Figure 3C) on the response of the lignan extraction yield were not considerable.

#### 2.2.4. Verification of Predictive Model

The optimal extraction conditions were predicted based on the RSM with the maximum extraction concentration of total lignans with Diol as the dispersant, 1:4 as the sample/dispersant ratio, and 85.30% MeOH elution for 9.93 mL. In order to verify the applicability of the quadratic equation in predicting the optimal response value, validation experiments were put into practice under the following adjusted conditions: Diol as the dispersant, a sample/dispersant ratio of 1:4 (800 mg), and 85% MeOH elution for 10 mL. The predictive value was 14.60 mg/g while the actual value of the total lignan extraction yield was 14.67 mg/g, demonstrating no significant difference between the actual value and the predictive value. Consequently, the extraction conditions obtained with RSM were accurate and reliable, and also had an application value reflecting the expected optimization.

### 2.3. Comparison with Traditional Extraction Method

The proposed method was compared with conventional pretreatment methods with different extraction operations for the measurement of lignans from complex materials to prove its potential application. According to the Chinese Pharmacopoeia Commission (2020), ultrasonic extraction has been utilized as a pretreatment method for *S. chinensis* [1]. Therefore, the difference between the developed Diol-based MSPD method and the ultrasonic extraction method was compared by extracting the target analytes in one batch of *S. chinensis*. The efficiency provided by MSPD (14.67 mg/g) was similar to that of the ultrasonic extraction method (14.63 mg/g), which demonstrated that the Diol-based MSPD method had nearly the same effectiveness as the method of the Chinese Pharmacopoeia Commission when extracting the targets in *S. chinensis.*

On the other hand, to further evaluate the performance of the currently developed MSPD method, a comparison method was carried out with some related parameters among the optimized methods and published methods by other scholars. In Table 4, it can be clearly observed that the items related to the Diol-based MSPD procedure, including extraction methods, sample amounts, type of solvent, and solvent volume, were either similar to or better than those from other published reports. To be specific, MSPD required fewer reagents and a shorter pretreatment time. The entire pretreatment process of the optimized MSPD method took about 20 min and the total amount of solvent used for each assay was 10 mL, which was less than the amounts used in smashing tissue extraction (38 mL) and pressurized liquid extraction (150 mL). In particular, MSPD does not require complex instruments and materials. Notably, there was a significant variation in lignan content in *S. chinensis* obtained from different cultivated areas, so not only can the difference in the reported amounts be ascribed to the extraction method, but it can also be ascribed to the nature of the sample. Comprehensively, considering the extraction efficiency, experimental time, and solvent consumption, the developed method of Diol-based MSPD was satisfactory for the rapid and effective determination of the major components in *S. chinensis*.

### 2.4. Application

To evaluate the practicality of the proposed method, 13 batches of *S. chinensis* samples obtained from different cultivated areas of China, including Liaoning, Jilin, Inner Mongolia, and Heilongjiang province, were analyzed. All 10 analytes were recognized in an HPLC chromatogram and the contents were calculated with external standard methods based on the respective calibration curves. The analytical results are revealed in Table 5; we observed that the contents of the investigated bioactive lignans in *S. chinensis* from different areas are noticeably diverse. It is supposed that variables such as geographical climate, cultivation year, harvest time, manufacturing procedure, and store conditions may be responsible for content variations in the bioactive markers. Therefore, it is valuable to institute an accurate quantitative analysis method for reasonable quality evaluation of valuable resources cultivated in different regions.

## 3. Materials and Methods

### 3.1. Chemicals and Reagents

Silica gel, HSO_3_-silica gel, C_18_, C_8_, Diol, XAmide, and Xion were supplied by ACChrom Tech (Beijing, China). Ultrapure water was processed with the MILLI-Q water purification system (Bedford, MA, USA). Acetonitrile and methanol of HPLC grade were purchased from Concord Technology (Tianjin, China), and all other chemicals were of analytical reagent grade and provided by Kermel (Tianjin, China). All reagents for HPLC were filtrated through 0.22 μm nylon syringe filter.

**Figure 4:**
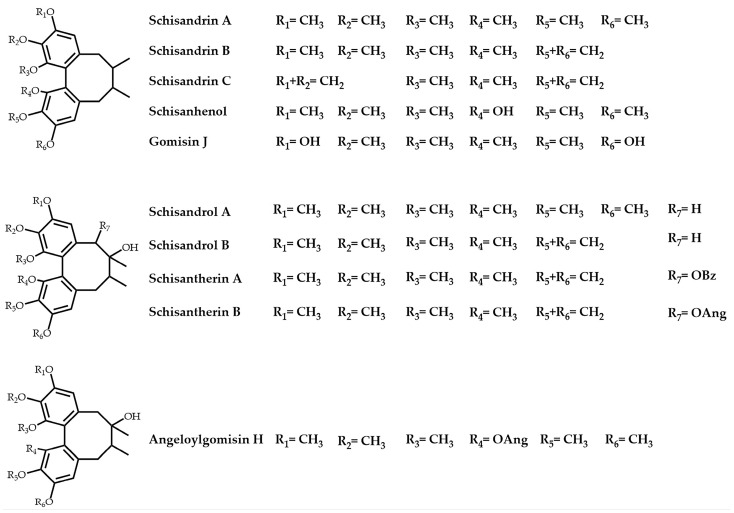
The structures of 10 analytes in *S. chinensis*.

The standards of schisandrin A and schisandrin B were purchased from the National Institutes for Food and Drug Control (Beijing, China). Schisantherin A, schisantherin B, schisanhenol, gomisin J, angeloylgomisin H, schisandrol A, schisandrol B, and schisandrin C were purchased from Chengdu Push Bio-technology Co., Ltd. (Chengdu, China). The purity of all analytes was more than 98%. The chemical structures of the 10 analytes are shown in Figure 4.

### 3.2. Plant Materials

Thirteen batches of *S. chinensis* were collected from Liaoning, Jilin, Heilongjiang, and Inner Mongolia provinces of China and identified by Prof. Jingbo Zhu (Dalian Polytechnic University, Dalian, China) through the Chinese Pharmacopoeia [1]. All samples were dried at 65 °C for 12 h, and pulverized by a versatile plant pulverizer and passed through a 20-mesh sieve, as preparation for later investigation.

### 3.3. Extraction of Lignans with MSPD

**Figure 5:**
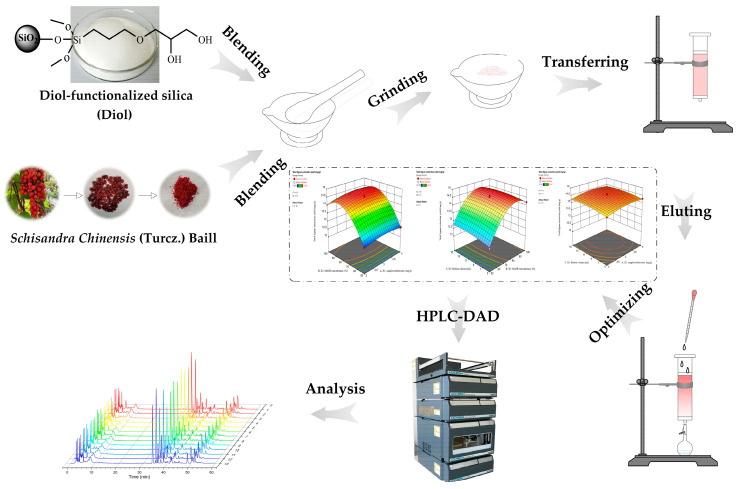
Schematic diagram of diol-functionalized silica (Diol)-based MSPD.

The *S. chinensis* powder (200 mg) and dispersant (800 mg) were precisely weighed, and transferred into the agate mortar in sequence. The mixture was ground for 5 min and transferred into a 12 mL polypropylene SPE cartridge in which a sieve plate was already prefilled at the bottom. Then, another thin layer of sieve plate was supplemented on the top of the sample mixture by light compression using a syringe plunger to get rid of undesirable channels. Later, the packed tube was rinsed with 10 mL of 85% methanol–water solution. The eluent was collected in a volumetric flask and made up to mark with methanol, and filtered through a 0.22 μm filter prior to analytical procedure. The schematic diagram of Diol-based MSPD method is shown in Figure 5.

### 3.4. Ultrasonic Extraction

The *S. chinensis* powder (200 mg) was accurately weighed and moved into a 25 mL volumetric flask and mixed with 20 mL of methanol. The mixture was ultrasonically extracted (40 KHz) for 20 min, and then adjusted to the mark with methanol. The final sample solution was passed through a 0.22 μm filter and submitted to the analytical system.

### 3.5. Determination of Lignans with HPLC-DAD

**Figure 6:**
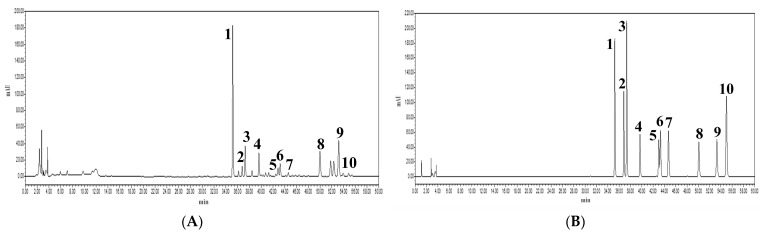
High-performance liquid chromatogram of extraction solution from *S. chinensis* (**A**) and standard compounds (**B**). Schisandrol A (1), gomisin J (2), schisandrol B (3), angeloylgomisin H (4), schisantherin A (5), schisantherin B (6), schisanhenol (7), schisandrin A (8), schisandrin B (9), schisandrin C (10).

The analyses were performed on an ACChrom S6000 HPLC system with ACChrom 3430 DAD (Acchrom Tech, Beijing, China). A FINDSIL C_18_ column (250 mm × 4.6 mm, 5 μm, Bomexc Tech, Dalian, China) was used for elution at 1 mL/min at a column temperature of 30 °C. The mobile phase included acetonitrile (solvent A) and pure water (solvent B) using a gradient elution: 10–50% A at 0–30 min, 50–60% A at 30–32 min, 60–85% A at 32–57 min, and 85–100% A at 57–60 min. The injection volume was 20 μL, and the detection wavelength was 250 nm. The number of lignan compounds in the fruit samples was measured by comparing the peak area of each lignans with the reference standards. Empower^TM^ software (https://www.empowersoftware.co.nz/ (accessed on 1 September 2023)) was used for data collection, integration, and analysis. Under the described chromatographic conditions, all the analytes, including standard solutions and samples prepared with the method of Diol-based MSPD, separated excellently (Figure 6).

### 3.6. Method Validation

To verify the performance of the method, the parameters, including linearity, LOD, LOQ, and precision, were evaluated by using standard solutions. Mixed stock solution containing 10 compounds was prepared by being dissolved in methanol. The calibration curves for each compound were obtained by diluting stock solutions at various concentrations, and linearity was evaluated in terms of coefficient of determination (R^2^). The LOD and LOQ were determined by measuring the ratio of signal to noise (S/N) for each compound. A series of solutions were injected until the S/N ratios reached 3 and 10 for LOD and LOQ, respectively. The intraday precision of the assay was determined through analysis of six replicate injections of samples under optimal conditions. The interday precision was assessed by analyzing the same solution on three successive days. The RSD (%) was used to evaluate the precision of the method.

### 3.7. Statistical Analysis

The experimental design and subsequent data analysis used to optimize the MSPD method were performed using Design-expert (Version 13, State-Ease Inc., Minneapolis, MN, USA).

## 4. Conclusions

In this study, a simple and rapid MSPD extraction method was proposed to simultaneously extract 10 lignans in *S. chinensis* and determine the contents through a common HPLC-DAD method. A unique hydrophilic stationary phase, Diol, was used as a dispersant in this MSPD process, which was superior to similar processes and highly efficient. This method is simple, time-saving, low-consumption, and precise. These properties render Diol-based MSPD coupled with HPLC-DAD as a viable replacement method to extract and analyze lignans in raw materials, bioactive formulations, and functional foods from *S. chinensis*.

## Figures and Tables

**Table 1:** Regression equation, linear range, limit of detection (LOD), limit of quantitation (LOQ), repeatability, precision, and recovery values of 10 analytes.

Analyte	Calibration Curve	R^2^	Linear Range (μg/mL)	LOD (μg/mL)	LOQ (μg/mL)	Precision (RSD%)	
						Intraday	Interday
Schisandrol A	Y = 25.6922X − 27.1625	0.9998	35–175	0.23	1.15	0.87	1.03
Gomisin J	Y = 47.0927X − 4.0778	0.9999	0.98–5.4	0.34	0.98	0.48	0.83
Schisandrol B	Y = 44.7520X − 12.7742	0.9999	3.4–20.4	0.30	0.90	0.80	0.98
Angeloylgomisin H	Y = 35.3511X + 0.2585	0.9998	7–42	0.22	1.49	0.81	0.95
Schisantherin A	Y = 31.6965X − 5.5645	0.9997	1.6–8	0.09	1.45	0.96	1.02
Schisantherin B	Y = 33.7828X − 3.3653	0.9999	2.07–10	0.43	2.07	0.94	1.15
Schisanhenol	Y = 19.7444X − 1.1312	0.9999	0.8–5.4	0.20	0.80	1.05	1.15
Schisandrin A	Y = 49.157X − 1.2122	0.9999	0.75–3.75	0.05	0.75	0.80	1.04
Schisandrin B	Y = 40.5254X − 19.6594	0.9997	7.5–45	0.35	0.70	0.61	0.80
Schisandrin C	Y = 60.0555X + 2.1668	0.9991	0.56–5.9	0.04	0.49	0.21	0.86

**Table 2:** Condition optimization and results of matrix solid-phase dispersion (MSPD).

No.	Levels			Y: Total Lignan Extraction Yield (mg/g)
	X_1_: Sample/Dispersant Ratio	X_2_: Methanol (MeOH) Concentration (%)	X_3_: Elution Volume (mL)	
1	1:3	75	10	14.45
2	1:2	75	5	14.04
3	1:3	75	10	14.54
4	1:4	100	10	14.24
5	1:3	100	5	14.20
6	1:4	75	15	14.37
7	1:3	75	10	14.46
8	1:2	100	10	14.03
9	1:3	75	10	14.40
10	1:2	50	10	12.57
11	1:3	50	5	12.41
12	1:3	75	10	14.60
13	1:2	75	15	14.20
14	1:3	100	15	14.29
15	1:4	75	5	14.29
16	1:3	50	15	12.75
17	1:4	50	10	12.62

**Table 3:** Analysis of mean square deviation of regression equation.

Source	Sum of Squares	df	Mean Square	F-Value	*p*-Value Prob > F	Significance
Model	9.53	9	1.06	182.02	<0.0001	Significant
X_1_	0.0578	1	0.0578	9.93	0.0161	
X_2_	5.14	1	5.19	882.80	<0.0001	
X_3_	0.0561	1	0.0561	9.64	0.0172	
X_1_·X_2_	0.0064	1	0.0064	1.10	0.3291	
X_1_·X_3_	0.0016	1	0.0016	0.2750	0.6162	
X_2_·X_3_	0.0156	1	0.0156	2.69	0.1453	
X_1_^2^	0.1028	1	0.1028	17.67	0.0040	
X_2_^2^	3.95	1	3.95	679.20	<0.0001	
X_3_^2^	0.0498	1	0.0498	8.56	0.0222	
Residual	0.0407	7	0.0058			
Lack of fit	0.0155	3	0.0052	0.8214	0.5462	Not significant
Pure error	0.0252	4	0.0063			
Cor total	9.57	16				

**Table 4:** Comparison of the proposed Diol-based MSPD method and other methods in the determination of lignans in *S. chinensis*.

No.	Extraction Method	Sample Amounts	Type of Solvent (Volume)	Extraction Parameter	Detection Method	Extracted Lignans	Total Lignans	Ref.
1	Smashing tissue extraction	2.00 g	75% EtOH (38 mL)	35 min Voltage: 180 V	HPLC-UV	Schisandrol A, schisantherin A, deoxyshcisandrin, schisandrin B, schisandrin C	13.89 mg/g	[35]
2	Ultrasound-assisted extraction	0.5 g	MeOH (20 mL)	40 min 60 °C, 70 kHz	HPLC-FLD	Schisandrol A, schisandrol B, schisandrin A, schisandrin B, schisandrin C	9.35–23.51 mg/g	[53]
3	Pressurized liquid extraction	1 g	MeOH (150 mL)	15 min, 125 °C	HPLC-DAD	Schisandrol A, gomisin J, schisandrol B, tigloylgomisin H, angeloylgomisin H, schisandrin A, γ-schisandrin, gomisin N, schisandrin C	17.21 mg/g	[54]
4	Diol-based MSPD	200 mg	85% MeOH (10 mL)	20 min	HPLC-DAD	Schisandrol A, gomisin J, schisandrol B, schisantherin A, schisantherin B, schisanhenol, schisandrin A, schisandrin B, schisandrin C, angeloylgomisin H	14.67 mg/g	Present work

**Table 5:** Contents of the 10 analytes of *Schisandra chinensis* (Turcz.) Baill. (*S. chinensis*) from different cultivated areas.

No.	Cultivation Areas	Analytes (mg/g)									
		Schisandrol A	Gomisin J	Schisandrol B	Angeloylgomisin H	Schisantherin A	Schisantherin B	Schisanhenol	Schisandrin A	Schisandrin B	Schisandrin C
1	Benxi, Liaoning	7.18 ± 0.11	0.24 ± 0.01	1.23 ± 0.03	1.03 ± 0.02	0.38 ± 0.01	0.62 ± 0.01	0.28 ± 0.01	0.46 ± 0.01	1.75 ± 0.04	0.16 ± 0.01
2	Tieling, Liaoning	9.30 ± 0.08	0.28 ± 0.01	1.15 ± 0.01	1.27 ± 0.01	0.72 ± 0.01	0.88 ± 0.01	0.59 ± 0.01	1.36 ± 0.01	2.24 ± 0.02	0.15 ± 0.01
3	Dandong, Liaoning	7.23 ± 0.08	0.27 ± 0.01	0.94 ± 0.01	0.97 ± 0.01	0.55 ± 0.01	0.69 ± 0.01	0.36 ± 0.01	1.06 ± 0.01	1.96 ± 0.01	0.12 ± 0.01
4	Yanbian, Jilin	7.96 ± 0.06	0.23 ± 0.01	1.08 ± 0.01	1.10 ± 0.01	0.55 ± 0.01	0.71 ± 0.01	0.36 ± 0.01	0.87 ± 0.01	1.92 ± 0.01	0.13 ± 0.01
5	Baishan, Jilin	8.38 ± 0.01	0.26 ± 0.01	1.15 ± 0.01	1.07 ± 0.01	0.68 ± 0.01	0.72 ± 0.01	0.35 ± 0.01	1.02 ± 0.01	1.96 ± 0.01	0.10 ± 0.01
6	Tonghua, Jilin	7.36 ± 0.04	0.30 ± 0.01	1.24 ± 0.01	1.01 ± 0.01	0.48 ± 0.01	0.62 ± 0.01	0.33 ± 0.01	0.74 ± 0.01	2.02 ± 0.02	0.17 ± 0.01
7	Elunchun, Inner Mongolia	8.42 ± 0.02	0.26 ± 0.01	1.13 ± 0.01	1.21 ± 0.01	0.62 ± 0.01	0.76 ± 0.01	0.40 ± 0.01	1.02 ± 0.01	2.21 ± 0.01	0.16 ± 0.01
8	Jiagedaqi, Heilongjiang	7.21 ± 0.17	0.24 ± 0.01	1.10 ± 0.03	1.01 ± 0.08	0.40 ± 0.01	0.69 ± 0.02	0.30 ± 0.01	0.53 ± 0.02	1.79 ± 0.05	0.14 ± 0.01
9	Yichun, Heilongjiang	5.85 ± 0.06	0.33 ± 0.01	1.29 ± 0.02	1.09 ± 0.01	0.30 ± 0.01	0.42 ± 0.01	0.27 ± 0.01	0.71 ± 0.01	2.61 ± 0.03	0.33 ± 0.01
10	Mudanjiang, Heilongjiang	9.24 ± 0.06	0.34 ± 0.01	1.07 ± 0.01	1.37 ± 0.05	0.59 ± 0.01	0.77 ± 0.01	0.39 ± 0.01	0.85 ± 0.01	2.42 ± 0.01	0.21 ± 0.01
11	Daxinganling, Heilongjiang	7.16 ± 0.01	0.43 ± 0.01	1.41 ± 0.01	1.00 ± 0.01	0.71 ± 0.01	0.27 ± 0.01	0.18 ± 0.01	0.62 ± 0.01	2.46 ± 0.01	0.10 ± 0.01
12	Heihe, Heilongjiang	10.85 ± 0.01	0.53 ± 0.01	1.75 ± 0.01	1.34 ± 0.01	0.88 ± 0.01	1.03 ± 0.01	0.64 ± 0.01	1.27 ± 0.01	2.21 ± 0.01	0.10 ± 0.01
13	Heihe, Heilongjiang	9.13 ± 0.01	0.45 ± 0.01	1.49 ± 0.01	1.11 ± 0.01	0.74 ± 0.01	0.86 ± 0.01	0.53 ± 0.01	1.05 ± 0.01	1.87 ± 0.01	0.08 ± 0.01

## Data Availability

The data that support the findings of this work are available from the corresponding author upon reasonable request.

## Sample Availability

Samples of the plant materials from *Schisandra chinensis* (Turcz.) Baill. are available from the authors.

## References

[1] Chinese Pharmacopoeia Commission (2020). Pharmacopoeia of the People’s Republic of China.

[2] Kopustinskiene D.M., Bernatoniene J. (2021). Antioxidant effects of *Schisandra chinensis* fruits and their active constituents. Antioxidants.

[3] Yang K., Qiu J., Huang Z.C., Yu Z.W., Wang W.J., Hu H.L., You Y. (2022). A comprehensive review of ethnopharmacology, phytochemistry, pharmacology, and pharmacokinetics of *Schisandra chinensis* (Turcz.) Baill. and *Schisandra sphenanthera* Rehd. et Wils. J. Ethnopharmacol..

[4] Chun J.N., Cho M., So I., Jeon J.H. (2014). The protective effects of *Schisandra chinensis* fruit extract and its lignans against cardiovascular disease: A review of the molecular mechanisms. Fitoterapia.

[5] Zhang C.N., Mao X., Zhao X., Liu Z., Liu B., Li H., Bi K.S., Jia Y. (2014). Gomisin N isolated from *Schisandra chinensis* augments pentobarbital-induced sleep behaviors through the modification of the serotonergic and GABAergic system. Fitoterapia.

[6] Zhu H.Y., Zhang L.N., Wang G.L., Xu Y.H., Gao Y.G., Zhang L.X. (2016). Sedative and hypnotic effects of supercritical carbon dioxide fluid extraction from *Schisandra chinensis* in mice. J. Food Drug Anal..

[7] Jiang Y.M., Fan X.M., Wang Y., Tan H., Chen P., Zeng H., Huang M., Bi H.C. (2015). Hepato-protective effects of six schisandra lignans on acetaminophen-induced liver injury are partially associated with the inhibition of CYP-mediated bioactivation. Chem.-Biol. Interact..

[8] Jung Y.S., Ok C.Y., Cho E.J., Park J.S., Lee S.K., Choi Y.W., Bae Y.S. (2013). Role of CXCR2 on the immune modulating activity of a-iso-cubebenol a natural compound isolated from the *Schisandra chinensis* fruit. Biochem. Biophys. Res. Commun..

[9] Zhao T., Feng Y., Li J., Mao R.W., Zou Y., Feng W.W., Zheng D.H., Wang W., Chen Y., Yang L.Q. (2014). *Schisandra* polysaccharide evokes immunomodulatory activity through TLR 4-mediated activation of macrophages. Int. J. Biol. Macromol..

[10] Mocan A., Schafberg M., Gianina Cris A., Rohn S. (2016). Determination of lignans and phenolic components of *Schisandra chinensis* (T urcz.) Baill. using HPLC-ESI-TOF-MS and HPLC-online TEAC: Contribution of individual components to overall antioxidant activity and comparison with traditional antioxidant assays. J. Funct. Foods.

[11] Mu X.L., Liu J.S., Li B., Wei X.P., Qi Y.D., Zhang B.G., Liu H.T., Xiao P.G. (2022). A comparative study on chemical characteristics, antioxidant, and hepatoprotective activity from different parts of *Schisandrae chinensis* Fructus. J. Food Process. Preserv..

[12] Li X.K., Ge J.M., Li M.Y., Deng S., Li J.R., Ma Y.C., Zhang J., Zheng Y.C., Ma L. (2022). Network pharmacology, molecular docking technology integrated with pharmacodynamic study to reveal the potential targets of Schisandrol A in drug-induced liver injury by acetaminophen. Bioorg. Chem..

[13] Zeng H., Jiang Y.M., Chen P., Fan X.M., Li D.S., Liu A.M., Ma X.C., Xie W., Liu P.Q., Gonzalez F.J. (2017). Schisandrol B protects against cholestatic liver injury through pregnane X receptors. Br. J. Pharmacol..

[14] Gmeiner A., Effenberger-Neidnicht K., Zoldáková M., Schobert R. (2011). A methyltitanocene complex of schisandrol A with high efficacy against multi-drug resistant cervix and breast carcinoma cells. Appl. Organomet. Chem..

[15] Lee D., Kim Y.M., Chin Y.W., Kang K.S. (2021). Schisandrol A exhibits estrogenic activity via estrogen receptor α-dependent signaling pathway in estrogen receptor-positive breast cancer cells. Pharmaceutics.

[16] Yan T.X., Sun Y.Y., Gong G.W., Li Y., Fan K.Y., Wu B., Bi K.S., Jia Y. (2019). The neuroprotective effect of schisandrol A on 6-OHDA-induced PD mice may be related to PI3K/AKT and IKK/IκBα/NF-κB pathway. Exp. Gerontol..

[17] Wu Y., Li Z.C., Yao L.Q., Li M., Tang M. (2019). Schisandrin B alleviates acute oxidative stress via modulating Nrf2/Keap1-mediated antioxidant pathway. Appl. Physiol. Nutr. Metab..

[18] Han J.B., Shi X.W., Du Y., Shi F.J., Zhang B., Zheng Z.X., Xu J.J., Jiang L.Q. (2019). Schisandrin C targets Keap1 and attenuates oxidative stress by activating Nrf2 pathway in Ang II-challenged vascular endothelium. Phytother. Res..

[19] Min X., Zhao L., Shi Y., Wang J., Lv H., Song X., Zhao Q., Zhao Q., Jing R., Hu J. (2022). Gomisin J attenuates cerebral ischemia/reperfusion injury by inducing anti-apoptotic, anti-inflammatory, and antioxidant effects in rats. Bioengineered.

[20] Ma R.J., Zhan Y.K., Zhang Y.M., Wu L.A., Wang X., Guo M. (2022). Schisandrin B ameliorates non-alcoholic liver disease through anti-inflammation activation in diabetic mice. Drug Dev. Res..

[21] Chen Y.Q., Kong Y., Wang Q.L., Chen J., Chen H., Xie H.H., Li L. (2021). Schisandrin B attenuates airway inflammation by regulating the NF-κB/Nrf2 signaling pathway in mouse models of asthma. J. Immunol. Res..

[22] Jin H.M., Wang Z., Gu Z.N., Wu J., Bai X.Q., Shao Z.X., Miao J.S., Wang Q.Q., Wang Q., Wang X.Y. (2018). Schisandrin B attenuates epidural fibrosis in postlaminectomy rats by inhibiting proliferation and extracellular matrix production of fibroblasts. Phytother. Res..

[23] Wang C.Q., Xu C., Fu X.L., Jiang Y.Y. (2020). Schisandrin B suppresses liver fibrosis in rats by targeting miR-101-5p through the TGF-β signaling pathway. Artif. Cells Nanomed. Biotechnol..

[24] Lee S.B., Kim C.Y., Lee H.J., Yun J.H., Nho C.W. (2009). Induction of the Phase II Detoxification Enzyme NQO1 in Hepatocarcinoma Cells by Lignans from the Fruit of *Schisandra chinensis* through Nuclear Accumulation of Nrf2. Planta Med..

[25] Liu H.T., Wu C.M., Wang S., Gao S.M., Liu J., Dong Z.Q., Zhang B.G., Liu M.Y., Sun X.B., Guo P. (2015). Extracts and lignans of *Schisandra chinensis* fruit alter lipid and glucose metabolism in vivo and in vitro. J. Funct. Foods.

[26] Kwan H.Y., Wu J.H., Su T., Chao X.J., Yu H., Liu B., Fu X.Q., Tse A.K.W., Chan C.L., Fong W.F. (2017). Schisandrin B regulates lipid metabolism in subcutaneous adipocytes. Sci. Rep..

[27] Sun D., Li Q., Li H., Li Y., Piao Z. (2014). Quantitative analysis of six lignans in fruits with different colours of *Schisandra chinensis* by HPLC. Nat. Prod. Res..

[28] Huang Y., Huang Z., Watanabe C., Wang L. (2019). Authentication of *Schisandra chinensis* and *Schisandra sphenantherae* in Chinese patent medicines by pyrolysis-gas chromatography/mass spectrometry and fingerprint analysis. J. Anal. Appl. Pyrolysis.

[29] Yu C., Xu Y., Wang M., Xie Z., Gao X. (2019). Application of characteristic fragment filtering with ultra high performance liquid chromatography coupled with high-resolution mass spectrometry for comprehensive identification of components in *Schisandrae chinensis* Fructus. J. Sep. Sci..

[30] Onay S., Hofer S., Ganzera M. (2020). Rapid analysis of nine lignans in *Schisandra chinensis* by supercritical fluid chromatography using diode array and mass spectrometric detection. J. Pharm. Biomed. Anal..

[31] Lambropoulou D.A., Albanis T.A. (2007). Methods of sample preparation for determination of pesticide residues in food matrices by chromatography-mass spectrometry-based techniques: A review. Anal. Bioanal. Chem..

[32] Wang M.C., Lai Y.C., Chang C.L. (2008). High throughput screening and antioxidant assay of dibenzo[*a,c*]cyclooctadiene lignans in modified-ultrasonic and supercritical fluid extracts of *Schisandra chinensis* Baill by liquid chromatography-mass spectrometry and a free radical-scavenging method. J. Sep. Sci..

[33] Sheng Y.H., Wang R., Wang Y.K., Li M.M., Liu F.X., Huang X., Chen C.B. (2022). Evaluation of multicomponent changes of *Schisandra chinensis* fruits with different drying process by UPLC-QQQ-MS-based targeted metabolomics analysis. J. Chem..

[34] Zhu M., Cao Y., Fan G.R. (2007). Microwave-assisted extraction and fingerprint studies of *Schisandra chinensis* (Turcz.) by high performance liquid chromatography and gas chromatography. J. Sep. Sci..

[35] Cheng Z.Y., Song H.Y., Yang Y.J., Zhou H.L., Liu Y., Liu Z.G. (2016). Smashing tissue extraction of five lignans from the fruit of *Schisandra chinensis*. J. Chromatogr. Sci..

[36] Mayya Razgonova A.Z.K.P., Kim E., Chernyshev V.A., Ercisli S., Cravotto G., Golokhvast K. (2020). Rapid mass spectrometric study of a supercritical CO_2_-extract from Woody Liana *Schisandra chinensis* by HPLC-SPD-ESI-MS/MS. Molecules.

[37] Barker S.A., Long A.R., Short C.R. (1989). Isolation of drug residues from tissues by solid dispersion. J. Chromatogr. Sci..

[38] Barker S.A. (2000). Matrix solid-phase dispersion. J. Chromatogr. A.

[39] Enríquez-Gabeiras L., Gallego A., Garcinuño R.M., Fernández-Hernando P., Durand J.S. (2012). Interference-free determination of illegal dyes in sauces and condiments by matrix solid phase dispersion (MSPD) and liquid chromatography (HPLC-DAD). Food Chem..

[40] Ding M.Y., Bai Y., Li J., Yang X.J., Wang H., Gao X.M., Chang Y.X. (2019). A Diol-based-matrix solid-phase dispersion method for the simultaneous extraction and determination of 13 compounds from *Angelicae Pubescentis* Radix by ultra high-performance liquid chromatography. Front. Pharmacol..

[41] Buszewski B., Noga S. (2012). Hydrophilic interaction liquid chromatography (HILIC)-a powerful separation technique. Anal. Bioanal. Chem..

[42] Huang G.H., Zeng W.C. (2010). Preparation and application of Diol-bonded monolithic ailica capillary column. Chin. J. Spectrosc. Lab..

[43] Zhu C., Dane A., Spijksma G., Wang M., van der Greef J., Luo G., Hankemeier T., Vreeken R.J. (2012). An efficient hydrophilic interaction liquid chromatography separation of 7 phospholipid classes based on a diol column. J. Chromatogr. A.

[44] Rosłon M., Jaworska M., Anuszewska E.L. (2022). Determination of glycerophospholipids in biological material using high-performance liquid chromatography with charged aerosol detector HPLC-CAD-a new approach for isolation and quantification. Molecules.

[45] Ares A.M., Bernal J., Janvier A., Toribio L. (2022). Chiral and achiral separation of ten flavanones using supercritical fluid chromatography. Application to bee pollen analysis. J. Chromatogr. A.

[46] Smiełowska M., Zabiegała B. (2019). Matrix solid-phase dispersion (MSPD) as simple and useful sample preparation technique for determination of polybrominated diphenyl ethers (PBDEs) in dust. Anal. Chim. Acta.

[47] Gritti F., Dos Santos Pereira A., Sandra P., Guiochon G. (2009). Comparison of the adsorption mechanisms of pyridine in hydrophilic interaction chromatography and in reversed-phase aqueous liquid chromatography. J. Chromatogr. A.

[48] Wang Q., Tong L., Yao L., Zhang P., Xu L. (2016). Fingerprinting of traditional Chinese medicines on the C18-Diol mixed-mode column in online or offline two-dimensional liquid chromatography on the single column modes. J. Pharm. Biomed. Anal..

[49] Xu J.J., Cao J., Peng L.Q., Cao W., Zhu Q.Y., Zhang Q.Y. (2016). Characterization and determination of isomers in plants using trace matrix solid phase dispersion via ultrahigh performance liquid chromatography coupled with an ultraviolet detector and quadrupole time-of-flight tandem mass spectrometry. J. Chromatogr. A.

[50] Cheng X.L., Qi L.W., Wang Q., Wan J.Y., Liu E.H., Li P. (2013). Highly efficient sample preparation and quantification of constituents from traditional Chinese herbal medicines using matrix solid-phase dispersion extraction and UPLC-MS/MS. Analyst.

[51] Chen X.F., Ding N., Zang H., Yeung H., Zhao R.S., Cheng C., Liu J.H., Chan T.W.D. (2013). Fe_3_O_4_@MOF core-shell magnetic microspheres for magnetic solid-phase extraction of polychlorinated biphenyls from environmental water samples. J. Chromatogr. A.

[52] Kalagatur N.K., Kamasani J.R., Siddaiah C., Gupta V.K., Krishna K., Mudili V. (2018). Combinational Inhibitory Action of *Hedychium spicatum* L. Essential Oil and γ-Radiation on Growth Rate and Mycotoxins Content of *Fusarium graminearum* in Maize: Response Surface Methodology. Front. Microbiol..

[53] Xia Y., Yang B., Liang J., Wang J., Kuang H. (2014). Simultaneous quantification of five dibenzocyclooctadiene lignans in *Schisandra chinensis* by HPLC separation and fluorescence detection. Anal. Methods.

[54] Lee H.J., Kim C.Y. (2010). Simultaneous determination of nine lignans using pressurized liquid extraction and HPLC-DAD in the fruits of *Schisandra chinensis*. Food Chem..

